# Evolution of ultrasound in giant cell arteritis

**DOI:** 10.3389/fmed.2022.981659

**Published:** 2022-10-03

**Authors:** Colm Kirby, Rachael Flood, Ronan Mullan, Grainne Murphy, David Kane

**Affiliations:** ^1^Department of Rheumatology, Tallaght University Hospital and Trinity College Dublin, Dublin, Ireland; ^2^Department of Rheumatology, Cork University Hospital and University College Cork, Cork, Ireland

**Keywords:** giant cell (temporal) arteritis, ultrasound, large vessel vasculitis, biomarkers, temporal artery biopsy

## Abstract

Ultrasound (US) is being increasingly used to diagnose Giant Cell Arteritis (GCA). The traditional diagnostic Gold Standard has been temporal artery biopsy (TAB), but this is expensive, invasive, has a false-negative rate as high as 60% and has little impact on clinical decision-making. A non-compressible halo with a thickened intima-media complex (IMC) is the sonographic hallmark of GCA. The superficial temporal arteries (STA) and axillary arteries (AA) are the most consistently inflamed arteries sonographically and imaging protocols for evaluating suspected GCA should include at least these two arterial territories. Studies evaluating temporal artery ultrasound (TAUS) have varied considerably in size and methodology with results showing wide discrepancies in sensitivity (9–100%), specificity (66–100%), positive predictive value (36–100%) and negative predictive value (33–100%). Bilateral halos increase sensitivity as does the incorporation of pre-test probability, while prior corticosteroid use decreases sensitivity. Quantifying sonographic vasculitis using Halo Counts and Halo Scores can predict disease extent/severity, risk of specific complications and likelihood of treatment response. Regression of the Halo sign has been observed from as little as 2 days to as late as 7 months after initiation of immunosuppressive treatment and occurs at different rates in STAs than AAs. US is more sensitive than TAB and has comparable sensitivity to MRI and PET/CT. It is time-efficient, cost-effective and allows for the implementation of fast-track GCA clinics which substantially mitigate the risk of irreversible blindness. Algorithms incorporating combinations of imaging modalities can achieve a 100% sensitivity and specificity for a diagnosis of GCA. US should be a standard first line investigation in routine clinical care of patients with suspected GCA with TAB reserved only for those having had a normal US in the context of a high pre-test probability.

## Introduction

Giant Cell Arteritis (GCA) is a vasculitis of large- and medium-sized vessels. It is the commonest idiopathic systemic vasculitis and incidence increases with age, predominantly affecting those aged > 70 years (1). Typical symptoms include headache, visual disturbance, jaw claudication and polymyalgia rheumatica (PMR). Prompt diagnosis and initiation of corticosteroids is key to prevent the most severe complications of stroke and/or irreversible blindness (2). The traditional gold standard for diagnosis involves performing a temporal artery biopsy (TAB) (3).

TAB has many shortcomings when assessing suspected cases of GCA. Not only is it costly and invasive, but it has repeatedly been shown to have a false negative rate as high as 60%, most likely due to inadequate sampling, skip lesions and pre-operative steroid use (4). Additionally, its impact on clinical decision-making is questionable. In recent years, the use of temporal artery ultrasound (TAUS) in assessing suspected GCA has increased considerably.

The definitions of what constitutes vasculitis on US are still evolving, as is our understanding of its true place not only in the diagnosis, but also in the long-term monitoring of GCA. Advances in technology have undoubtedly contributed hugely to this growing body of knowledge and we suggest where future innovations might lead to. We also compare TAUS to other imaging modalities in GCA and discuss how TAUS is currently utilized in routine clinical care with reference to current international guidelines. Lastly we describe our current understanding of the reliability and applicability of TAUS and suggest where US may ultimately be incorporated into a diagnostic algorithm for GCA.

## Impact of TAB on clinical decisions

TAB still has high value as a diagnostic test due to specificity of 100% for a diagnosis of GCA. However, given the high false-negative rate it is clear that many, if not the majority, of GCA patients are diagnosed based on clinical criteria despite the presence of a negative TAB result. A number of studies have examined the impact of TAB results on clinical decisions within this context. In one retrospective cohort of 290 patients in whom GCA was suspected with a subsequent negative diagnostic test (147 of whom had a negative bilateral TAUS and 143 of whom had a negative unilateral TAB), there was no between-group difference in the number of patients who had steroids discontinued, despite further stratification accounting for pre-test probability of having GCA. Additionally, there was no between-group differences noted in adverse outcomes (including blindness) or number of alternative diagnoses considered. These findings suggest that TAUS serves the same purpose as TAB but without the associated procedural risks while other large retrospective cohorts have shown that 41–87% of those with negative biopsies have corticosteroid therapy continued anyway (5–7). Thus, while most TABs that are performed are negative, in most cases negative TABs have no impact on clinical decision-making. Importantly, data suggests that incorporating TAUS into the workup for suspected GCA increases the positive yield of TABs from 8.5 to 24% with an associated 38% reduction in the number of TABs being performed overall and with a substantial cost-saving (8, 9).

## Defining the presence of vasculitis on ultrasound

**Figure 1:**
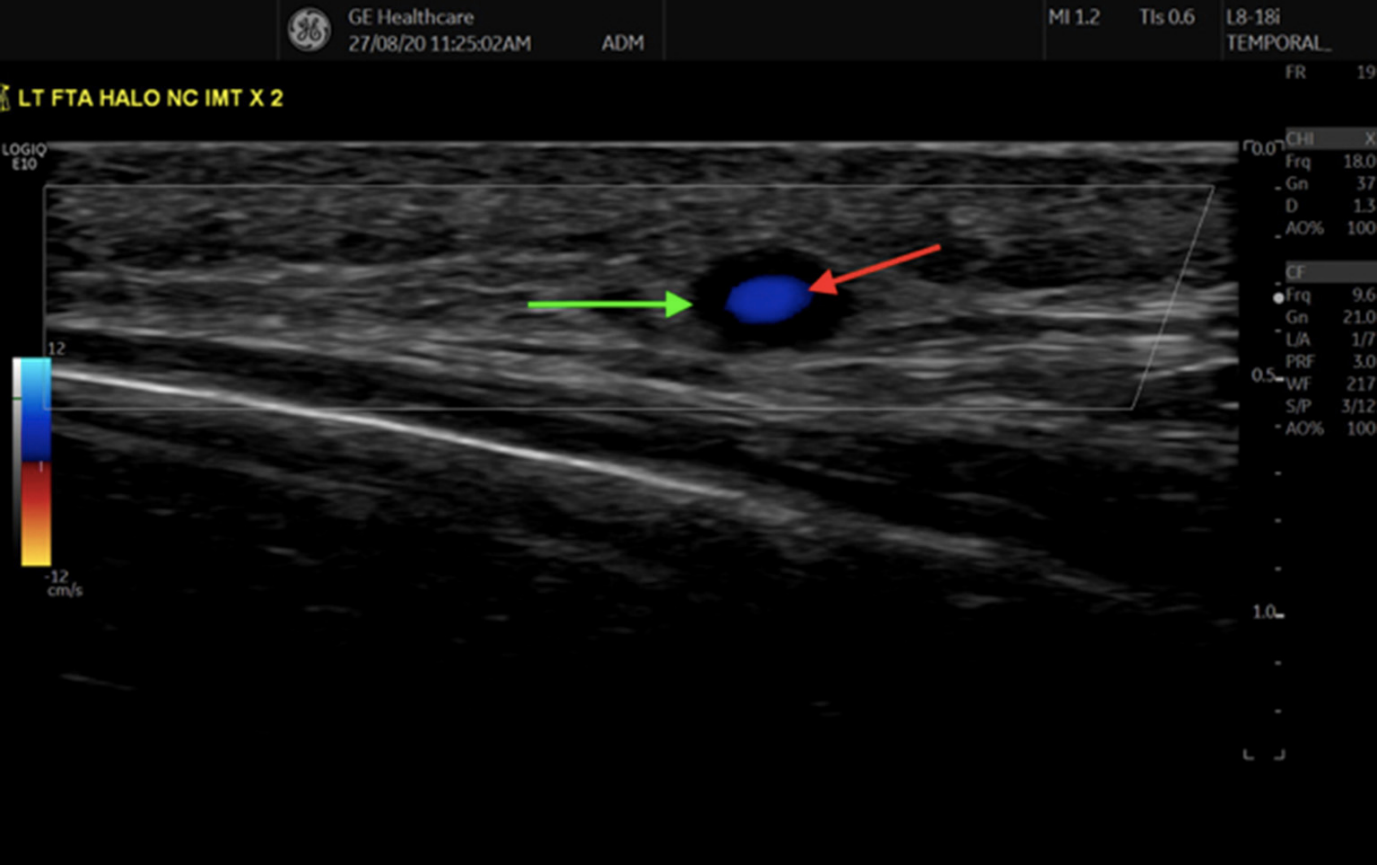
Transverse view of the frontal branch of the Superficial Temporal Artery, demonstrating a halo sign, as indicated by the anechoic region (green arrow) surrounding the inner Doppler (red arrow) signal.

In 1995 *Schmidt* first described, what still remains to this day, the cardinal sonographic hallmark of vasculitis- “The Halo Sign” (Figure 1) (10). It describes a sonographically hypoechoic ring of inflamed, oedematous vessel wall, surrounding the lumen of an artery. In a prospective study of 30 patients with clinically diagnosed GCA, confirmed by two independent rheumatologists, 22/30 had a Halo Sign identified in their superficial temporal arteries (STA), bilaterally in 17, and the rate of agreement between the two sonographers was 100%. No Halo Sign was identified in the 82 patients who had GCA excluded on clinical grounds (11).

**Figure 2:**
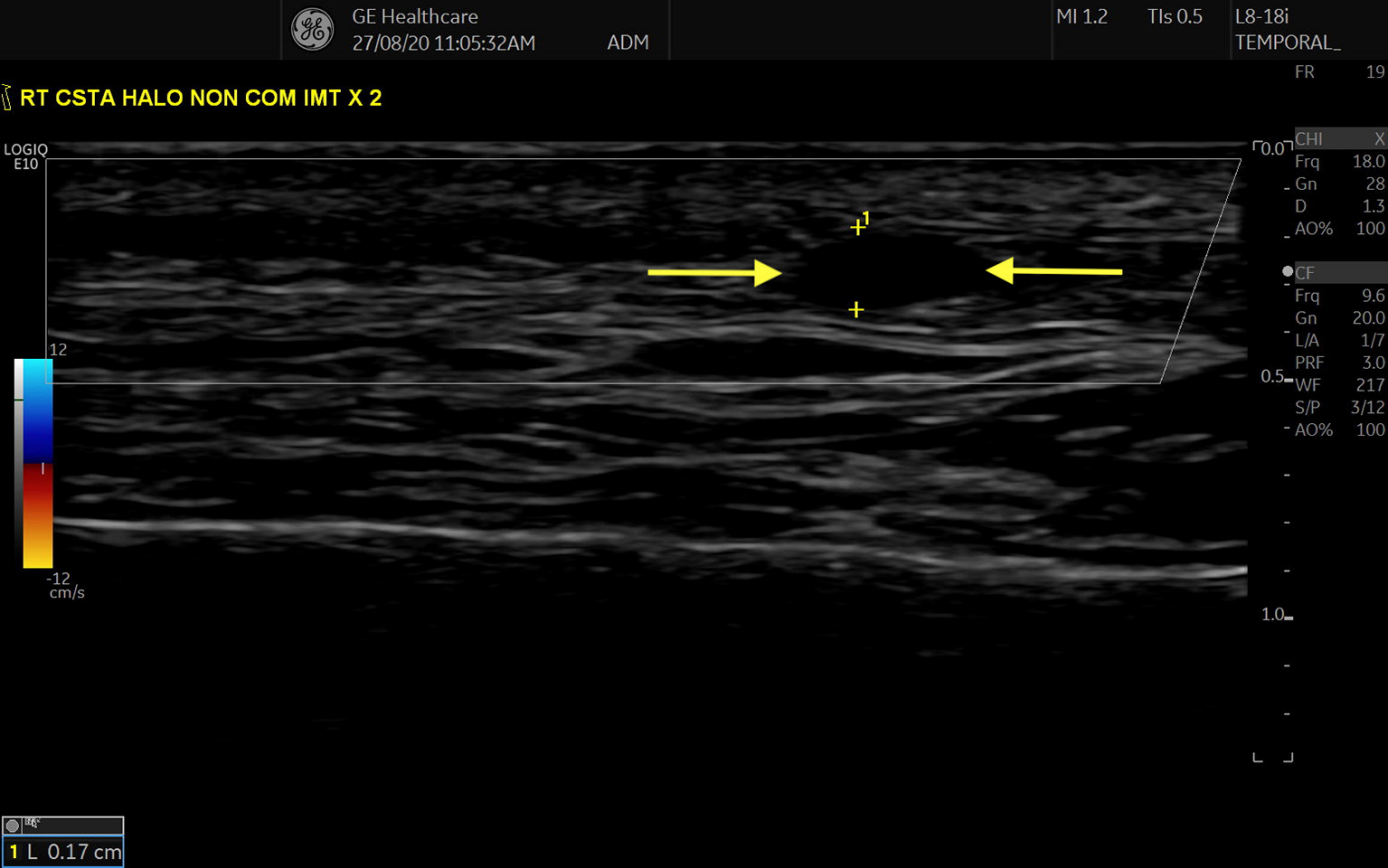
“Compression” sign in STA, transverse view. Hypoechoic/ anechoic region between two parallel hyperechoic lines (adventitia) represents an oedematous Intima-Media Complex (region between two yellow arrows).

In 2012, a new sonographic hallmark of vasculitis was reported: “The Compression Sign” (Figure 2). In a prospective study of 80 suspected cases of GCA (43 ultimately diagnosed as GCA based an ACR criteria), all participants had bilateral TAUS performed, examining for the presence of Halo Sign and/or Compression Sign. The Compression Sign was defined as persistent visibility of the STA despite transducer-imposed arterial compression (i.e., persistent contrasting echogenicity between vessel wall and surrounding tissue). Three physician-sonographers were involved in scanning and were blinded to the clinical details of the case. Interestingly, the Halo Sign and Compression Sign were both observed in 34/43 GCA patients and both signs were absent in all patients in the non-GCA group, showing a sensitivity and specificity of 79 and 100% respectively, for both signs in diagnosing GCA (12, 13). In 2018, the OMERACT LVV US working group defined the Halo and Compression Signs as the most significant sonographic abnormalities of GCA with inter-rater agreements of 91–99% and mean kappa values of 0.83–0.98 for both inter-rater and intra-rater reliabilities. The group defined the Halo Sign as “homogenous, hypoechoic wall thickening, well delineated toward the luminal side, visible both in longitudinal and transverse planes, most commonly concentric in transverse scans.” The Compression Sign was defined as being assessed “by applying pressure *via* the transducer until the lumen of the temporal artery occludes and no arterial pulsation remains visible” (14).

**Figure 3:**
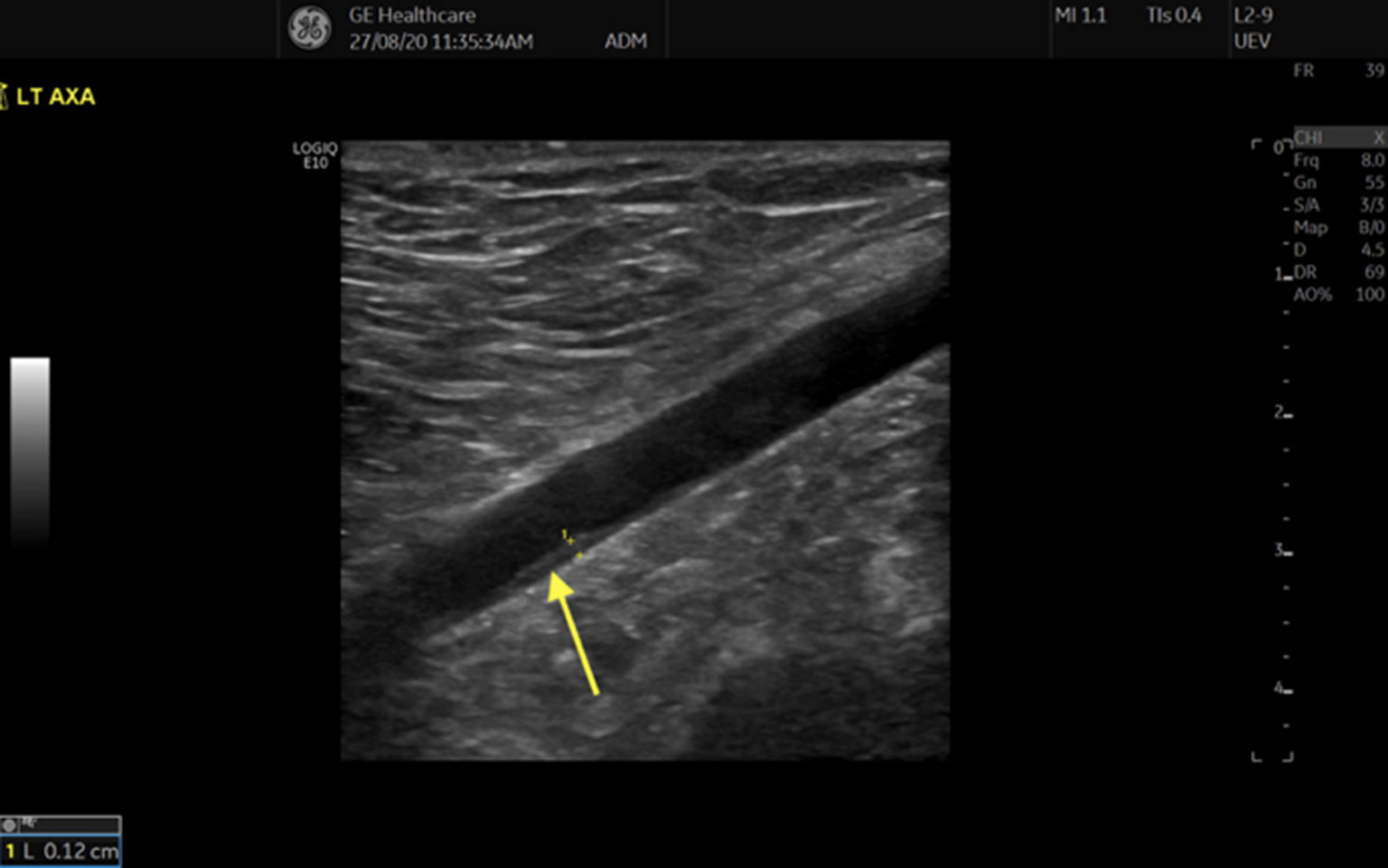
“Slope” sign in axillary artery vasculitis (yellow arrow).

More recently, the “Slope Sign” of axillary artery (AA) vasculitis has been described. This sign describes a long, thickened segment of inflamed arterial wall that slides down to a normal intima-media structure (double line) (Figure 3). In 214 patients referred to a fast-track GCA clinic, 81 were diagnosed with GCA, 23 of whom had axillary vasculitis. The slope sign was observed in all patients with AA vasculitis (15, 16).

## Normal vs. abnormal intima-media thickness

In 2017, normal cut-off values for intima-media thickness (IMT) of arteries involved in GCA were first published. IMT measurements of the STAs, facial arteries and AAs in 40 new GCA cases and 40 controls were obtained, with the gold standard being a clinical diagnosis of GCA. The cut-off values with sensitivities and specificities of the various arterial segments for a diagnosis of GCA are outlined in Table 1 (17). A recent study looked at 101 patients aged > 50 years, without a diagnosis of GCA or PMR, but with varying degrees of perceived cardiovascular (CV) risk. US of STAs and AAs were performed on all and notably, in those deemed to have high/very high CV risk, mean IMT was greater than in those with moderate/low risk both in STAs and AAs. IMT was greater than standard normal cut-off values in at least one artery in 10.1% of patients, 80% of whom had very high/ high CV risk (18).

**Table 1:** Cut-off values for distinguishing vasculitic artery from normal artery in suspected cases of GCA with sensitivities and specificities for a clinical diagnosis of GCA (17).

**Artery**	**IMT** **cut-off (mm)**	**Sensitivity**	**Specificity**
Common Superficial Temporal Artery (STA)	0.42	100%	100%
Frontal branch of STA	0.34	100%	100%
Parietal branch of STA	0.29	97.2%	98.7%
Facial artery	0.37	87.5%	98.8%
Axillary artery	1.0	100%	100%

Thus, while early sonographic definitions of vasculitis included the presence of vessel stenosis and occlusions, the current standard is to diagnose vasculitis based on the presence of a Halo Sign, a non-compressible artery (Compression Sign) and a thickened intima-media complex (IMC). The precision of these definitions remains a constant process of refinement and further research is needed in this field to further specify normal/abnormal IMT values. Additionally, false-positive ultrasounds can occur, as demonstrated in a cohort of 305 patients in whom TAUS confirmed the presence of a Halo Sign, but 14 of whom ultimately had a variety of diagnoses other than GCA (19).

## Distribution of pathology in GCA on US

Up to 2002, the frequency and location of peripheral arterial sonographic changes in GCA was unknown. In 10/33 GCA patients in one study, a Halo Sign could be demonstrated in peripheral arteries but importantly, sonographic vasculitis was consistently present in STAs and/or AAs if also present elsewhere (21). Additionally, it has been demonstrated that performing AAUS increases the diagnostic yield for large-vessel GCA with a detection-rate of 98 vs. 62% for TAUS alone, while asymptomatic abdominal aortic aneurysms may be detected in 33% of biopsy-proven GCA cases on US despite no clinical evidence of same (22–25). Other studies have consistently shown varying degrees of involvement of occipital, vertebral, carotid and femero-popliteal arteries (26–30). However, STA and AA are the most consistently inflamed arteries sonographically and imaging protocols for evaluating suspected GCA should include at least these two arterial territories.

## Temporal artery ultrasound in diagnosing GCA

Over the past 25 years, many studies have examined the diagnostic performance of TAUS against that of biopsy and clinical criteria (Table 2). In 2005, Karassa et al. undertook the first meta-analysis including all studies of >4 patients, which investigated the sensitivity and specificity of TAUS in GCA, using TAB or ACR classification criteria as the gold standard. Twenty-three studies of 2,036 patients demonstrated a weighted sensitivity and specificity of the Halo sign of 69 and 82%, respectively, compared with biopsy, and 55 and 94%, respectively, compared with ACR criteria. The studies included were mostly small with heterogenous methodology but they did show that in the presence of a low pre-test probability of GCA, a negative ultrasound can help out-rule the disease (31). A second meta-analysis was published in 2010 specifically examining the Halo sign and included eight studies of 575 patients (204 with GCA). Unilateral Halo sign achieved an overall sensitivity and specificity of 68 and 91%, respectively for GCA. The odds of having GCA in patients with a Halo Sign vs. in those without (pooled diagnostic odds ratio) was 34 (32). A systematic review by *Ball* looked at trials comparing TAUS and TAB and included 17 homogenous studies of 998 patients. When the sonographic halo was compared with TAB, the sensitivity was 75% and the specificity was 83%, leading the authors to conclude that TAUS was relatively accurate for diagnosing GCA and had promise as a first-line investigation, perhaps with TAB being reserved only for those with a normal US (33).

**Table 2:** Meta-analyses and systematic reviews relating to TAUS.

**Author**	**Year**	**Sample size-total (GCA)**	**Reference standard**	**US definition of vasculitis**	**Sensitivity**	**Specificity**
Karassa	2005	2,036	Clinical	Halo	55%	94%
			Biopsy	Halo	69%	82%
Arida	2010	575 (204)	Clinical	U/L Halo	68%	91%
Ball	2010	998	Biopsy	Halo / stenosis /occlusion	75%	83%
Duftner	2018	605 (605)	Clinical	Halo	77%	96%
			Clinical	MRI Cranial Arteries	73%	88%
			Clinical/ Biopsy	PET-CT	67–77%	66–100%
Rinagel	2019	20 studies	Biopsy	Halo	68%	81%

The seminal TABUL study was published in 2016 and showed that US was more sensitive and cost effective than TAB in investigating suspected GCA and importantly, the sensitivity of TAB was only 39% vs. previously published figures of >80%. In this prospective, multicentre study of 381 patients (257 with a reference standard clinical diagnosis of GCA, 124 without), all patients underwent US followed by TAB within 7 days of commencing treatment for GCA. 101 patients and 162 patients had positive TAB and US, respectively, with concordant results in 70% (kappa value 0.35). The sensitivities and specificities of biopsy and ultrasound were 39 and 100%, and 54 and 81%, respectively. Positive biopsy rate fell after 3 days of high-dose glucocorticoids whereas US abnormalities regressed within 4 days [a finding which concurred with those of a number of other studies highlighting the prompt regression of Halo Sign in those on corticosteroids (11, 34, 35)]. Of note, the authors demonstrated that a strategy of combining clinical assessment with US results was substantially cheaper (£485 per patient) than combining clinical assessment with biopsy (4).

Many studies therefore, have interrogated US as a diagnostic tool in GCA over the past two decades but have varied considerably in size and methodology with results showing wide discrepancies in sensitivity (9–100%), specificity (66–100%), positive predictive value (36–100%) and negative predictive value (33–100%) of US for a clinical or histological diagnosis of GCA. Most of the conflicting results are likely related to heterogenous methodologies, variances in sonographer technique and advances in ultrasound technology. The ever-expanding literature in this field has also shown us that the presence of bilateral halos increases sensitivity of US as does the incorporation of pre-test probability, while duration of prior corticosteroid use correlates inversely with likelihood of having a positive scan. A prospective study at Southend University Hospital assessing the validity of their pre-test probability score will be published in late 2022 (36).

## TAUS in monitoring GCA

Early studies evaluating the role of US in GCA disease-monitoring seemed to agree that Halo regression occurred within 3–4 weeks of initiating treatment (11, 21, 37–40). Later studies however, presumably due to improvements in US technology, identified halo persistence as late as 6 months after commencing immunosuppressive treatment for GCA (41–43). It is also notable that halo regression appears to occur more quickly in STA than AA, in those with relapsing vs. new-onset disease, in those who achieve clinical remission earlier, and in those with fewer STA branches affected at baseline (44–46). By contrast however, it has been demonstrated that there is no difference in relapse rate/steroid consumption between those with and without wall-thickening regression (47). In 2021, a prospective study evaluated the role of US in monitoring GCA in a cohort of 49 patients. The number of arterial segments with halo and the maximal IMT were measured at weeks 1, 3, 6, 12 and 24 and showed significant differences at all time points in STAs and after 6 weeks in AAs. Higher halo numbers/thickness correlated with inflammatory markers, cumulative steroid dose and lower likelihood of achieving remission with no such associations seen for AA halo. In cases of relapse, 16/17 cases had increased halo IMT compared to last measured value (48). However, no reliable conclusions can be drawn regarding the use of US in monitoring GCA based on available data.

## Development of an US score in GCA

A sub-study of the TABUL cohort demonstrated that an US score incorporating maximal IMT and bilaterality of STA/AA halos was useful for predicting likelihood for positive TAB but not for predicting clinical outcome at 6 months (49). More recently, Halo Counts (HC, number STA/AA branches with Halo) and Halo Scores (HS, composite of number and size of halos, Table 3) have been described. Both have shown a high degree of sensitivity for a clinical diagnosis of GCA (area under ROC curve 0.892 and 0.921) and strong associations with degrees of systemic inflammation and likelihood of ocular complications. In addition, the scores correlate positively with likelihood of having a subsequent positive TAB and they appear to be unaffected by cumulative steroid dose over the first week of treatment (50). These scores have so far been validated in one inception cohort for diagnosis but research is ongoing to assess their utility in monitoring disease activity long-term (20).

**Table 3:** Each branch is assigned a score based on the maximal intima-media thickness (IMT) identified in that branch.

**Halo score**	**Common STA**	**Frontal STA**	**Parietal STA**	**Axillary Artery**
0	0–0.3	0–0.1	0–0.2	0–0.5
1	0.31–0.4	0.11–0.2	0.21–0.3	0.51–0.6
2	0.41–0.5	0.21–0.3	0.31–0.4	0.61–0.89
3	0.51–0.79	0.31–0.49	0.41–0.59	0.9–1.5
4	≥ 0.8	≥ 0.5	≥ 0.6	≥ 1.6

IMT ranges (in millimeters) and their corresponding scores are outlined. Values for axillary arteries are multiplied by three to account for it having fewer branches. The scores are added to give a Total Halo Score, with a maximum value of 48. Halo scores are evaluated on serial scans to assess for wall-thickness regression. STA, Superficial Temporal Artery (50).

## TAUS in predicting GCA phenotype and prognosis

In a number of studies, STA involvement without AA involvement is predictive of ocular disease with a stronger association being seen for those with bilateral halos (50–54). Involvement of both STAs and AAs infers a significantly higher risk of relapse and a more frequent requirement for steroid-sparing agents relative to those patients with either isolated cranial or isolated upper limb GCA with a similar association being seen for higher baseline HC and HS (48, 55).

## Comparison of ultrasound with other imaging modalities in GCA

MRI has the resolution to accurately depict vessel wall thickening and oedema using contrast agents. While primarily used for large vessels, recent protocols specifically for STAs have shown promise but sensitivities for a diagnosis of GCA vary widely (33.3–69% sensitivity, 87.5–91% specificity) and a combination of clinical examination and US have shown higher sensitivities (66.7 and 77.7%) and specificities (100%) relative to MRI (56, 57). Additionally, the sensitivity of baseline US and MRI of STAs for diagnosing GCA reduce rapidly with corticosteroid treatment. With TAB as the reference gold-standard, the respective sensitivities of MRI and US in a cohort of 59 suspected cases of GCA were as follows: 90 and 92% (when scanned within 1^st^ day after steroid initiation), 77.8 and 80% (when scanned within 2–4 days after steroid initiation) and 80 and 50% (when scanned more than 4 days after steroid initiation) (58). Notably, other data have shown no statistical difference between US and MRI for detecting superficial cranial vessel vasculitis while US appears to detect vasculitic change more frequently than MRI both in those with new-onset disease and in those with chronic disease in the axillary, subclavian and carotid arteries (59). Multiple studies have also evaluated PET/CT relative to US in diagnosing GCA. PET/CT has shown greater sensitivity than US for identifying vertebral artery lesions but comparable sensitivity for diagnosing large-vessel disease, although abnormalities are often incongruous within single vascular regions (60–62).

## TAUS in routine clinical practice

While US is clearly a very useful clinical tool in rheumatology, as recently as 2014 only 1% of its use among rheumatologists was for the purpose of diagnosing vasculitis while 74–94% of rheumatologists prefer TAB over TAUS as a confirmatory test for GCA (63–65). However, since the publication of updated EULAR guidelines on imaging in LVV in 2018, its use has increased considerably, as reported by De Miguel et al., citing data from the Spanish ARTESER registry.

A number of European rheumatology centers have equipped themselves with the technology and expertise to operate Fast-track GCA clinics, which consist of same-day TAUS and initiation of treatment. The relative risk of permanent blindness in the GCA patients diagnosed through the Fast-track clinic is 88% lower compared with those diagnosed by the conventional route with a shorter mean duration of inpatient care by 3 days (66). The effectiveness of standardized training programmes for TAUS has shown excellent inter-reader reliability. In a study of 112 GCA patients who has vascular US (VUS) performed by five sonographers who underwent standardized training, an interobserver agreement of 95–96% with mean kappa values of 0.88–0.92 (95% CI 0.78 to 0.99) were achieved (67).

## Past and present innovations in US technology

Recently, very-high resolution ultrasound (VHRU, 55 MHz) has been shown to non-invasively and reliably, define the thickness of the arterial intima layer. In 37 patients who had negative TAB, intimal thickening (IT >0.06 mm on histology) could be identified as a “four-line pattern” in VHRU with a sensitivity and specificity of 96.3 and 100% respectively and excellent agreement between histologic and VHRU IT measurement (68). Recently, in a proof-of-concept study in 24 GCA patients, contrast-enhanced US (CEUS) of large vessels had a sensitivity and specificity of 91.7 and 100% for detecting active LVV (69). It provides detailed images of lumen-to-vessel wall border and abnormalities correlate well with those seen on FDG-PET (70, 71). Most significantly however, Roncato et al. have described an automated image analysis tool for diagnosis of GCA using artificial intelligence (AI) algorithms. They reported on a retrospective cohort of 137 patients with suspected GCA who had VUS performed and labelled with VIA software. They obtained a sensitivity of 60% and specificity of 95% for their test set (72). Yet, while inter-rater agreements for US are high, it is an inherently subjective test with interpretation relying upon sonographer expertise/ experience. Incorporation of AI algorithms will provide more objectivity and standardization of US between individuals/centers and, we expect, eliminate the disparities between study results that we have observed to date.

## Current recommendations for the use of US in GCA

In 2018, EULAR issued its first guidance document on the use of imaging in LVV, including ultrasound, with a new taskforce expected to update these recommendations in 2023 (73). In addition to some technical specifications, they state the following:

1. In patients with suspected GCA, an early imaging test is recommended to complement the clinical criteria for diagnosing GCA, assuming high expertise and prompt availability of the imaging technique.
2. In patients in whom there is a high clinical suspicion of GCA and a positive imaging test, the diagnosis of GCA may be made without an additional test (biopsy or further imaging). In patients with a low clinical probability and a negative imaging result, the diagnosis of GCA can be considered unlikely.
3. Ultrasound of temporal ± axillary arteries is recommended as the first imaging modality in patients with suspected predominantly cranial GCA. A non-compressible “Halo” sign is the ultrasound finding most suggestive of GCA.

The BSR has also issued recommendations for evaluating and managing GCA (74). They strongly recommended using a confirmatory diagnostic test, either TAUS, TAB, or both and they stress the importance of considering the pre-test probability prior to initiating investigations.

US should be a standard first line investigation in routine clinical care of patients with suspected GCA with TAB perhaps reserved for those only having had a normal US in the context of a high pre-test probability. It is more sensitive and cost-effective than TAB with an estimated saving of approximately €500 per patient and performs as well as MRI and PET/CT with the added benefit of easier access and lower relative cost when compared to those two investigations. Importantly however, it has been shown that algorithms incorporating combinations of imaging modalities can achieve a 100% sensitivity and specificity (62, 75). Moving forward, it is likely that such algorithms will become the Gold Standard in diagnosing GCA, rather than clinicians having to rely upon one specific test.

## Author contributions

All authors listed have made a substantial, direct, and intellectual contribution to the work and approved it for publication.

## Conflict of interest

The authors declare that the research was conducted in the absence of any commercial or financial relationships that could be construed as a potential conflict of interest.

## Publisher's note

All claims expressed in this article are solely those of the authors and do not necessarily represent those of their affiliated organizations, or those of the publisher, the editors and the reviewers. Any product that may be evaluated in this article, or claim that may be made by its manufacturer, is not guaranteed or endorsed by the publisher.

## References

[1] Duftner C Dejaco C Sepriano A Falzon L Schmidt WA Ramiro S. Imaging in diagnosis, outcome prediction and monitoring of large vessel vasculitis: a systematic literature review and meta-analysis informing the EULAR recommendations. RMD open. (2018) 4:e000612. 10.1136/rmdopen-2017-000612 29531788 PMC5845406

[2] Salvarani C Pipitone N Versari A Hunder GG. Clinical features of polymyalgia rheumatica and giant cell arteritis. Nat Rev Rheumatol. (2012) 8:509–21. 10.1038/nrrheum.2012.97 22825731

[3] Hall S Persellin S Lie JT O'Brien PC Kurland LT Hunder GG. The therapeutic impact of temporal artery biopsy. Lancet. (1983) 2:1217–20. 10.1016/S0140-6736(83)91269-2 6139569

[4] Luqmani R Lee E Singh S Gillett M Schmidt WA Bradburn M. The role of ultrasound compared to biopsy of temporal arteries in the diagnosis and treatment of giant cell arteritis (TABUL): a diagnostic accuracy and cost-effectiveness study. Health Technol Assess. (2016) 20:1–238. 10.3310/hta20900 27925577 PMC5165283

[5] Alberts MS Mosen DM. Diagnosing temporal arteritis: duplex vs. biopsy. QJM: Monthly Journal of the Association of Physicians. (2007) 100:785–9. 10.1093/qjmed/hcm103 18089544

[6] Deyholos C Sytek MC Smith S Cardella J Orion KC. Impact of temporal artery biopsy on clinical management of suspected giant cell arteritis. Ann Vasc Surg. (2020) 69:254–60. 10.1016/j.avsg.2020.06.012 32554192

[7] Bowling K Rait J Atkinson J Srinivas G. Temporal artery biopsy in the diagnosis of giant cell arteritis: Does the end justify the means? Ann Med Surg. (2012). 20:1–5. 10.1016/j.amsu.2017.06.020 28663795 PMC5479941

[8] Cristaudo AT Mizumoto R Hendahewa R. The impact of temporal artery biopsy on surgical practice. Ann Med Surg. (2012) 11:47–51. 10.1016/j.amsu.2016.09.004 27699002 PMC5037119

[9] Alberts M. Temporal arteritis: improving patient evaluation with a new protocol. Perm J. (2013) 17:56–62. 10.7812/TPP/12-067 23596371 PMC3627791

[10] Schmidt WA Kraft HE Völker L Vorpahl K Gromnica-Ihle EJ. Colour Doppler sonography to diagnose temporal arteritis. Lancet. (1995) 345:866. 10.1016/S0140-6736(95)93005-1 7898257

[11] Schmidt WA Kraft HE Vorpahl K Völker L Gromnica-Ihle EJ. Color duplex ultrasonography in the diagnosis of temporal arteritis. N Engl J Med. (1997) 337:1336–42. 10.1056/NEJM199711063371902 9358127

[12] Aschwanden M Daikeler T Kesten F Baldi T Benz D Tyndall A. Temporal artery compression sign–a novel ultrasound finding for the diagnosis of giant cell arteritis. Ultraschall in der Medizin. (2013) 34:47–50. 10.1055/s-0032-1312821 22693039

[13] Aschwanden M Imfeld S Staub D Baldi T Walker UA Berger CT. The ultrasound compression sign to diagnose temporal giant cell arteritis shows an excellent interobserver agreement. Clin Exp Rheumatol. (2015) 33( 2 Suppl 89):S-113–5. 26016760

[14] Chrysidis S Duftner C Dejaco C Schäfer VS Ramiro S Carrara G. Definitions and reliability assessment of elementary ultrasound lesions in giant cell arteritis: a study from the OMERACT Large Vessel Vasculitis Ultrasound Working Group. RMD Open. (2018) 4:e000598. 10.1136/rmdopen-2017-000598 29862043 PMC5976098

[15] Milchert M Brzosko M Bull Haaversen A Diamantopoulos AP. Correspondence to ‘Slope sign': a feature of large vessel vasculitis? Ann Rheum Dis. (2019) 80:e198. 10.1136/annrheumdis-2019-216601 31776115

[16] Dasgupta B Smith K Khan AAS Coath F Wakefield RJ. ‘Slope sign': a feature of large vessel vasculitis? Ann Rheum Dis. (2019) 78:1738. 10.1136/annrheumdis-2019-216213 31519653

[17] Schäfer VS Juche A Ramiro S Krause A Schmidt WA. Ultrasound cut-off values for intima-media thickness of temporal, facial and axillary arteries in giant cell arteritis. Rheumatology. (2017) 56:1479–83. 10.1093/rheumatology/kex143 28431106

[18] Martire MV Cipolletta E Di Matteo A Di Carlo M Jesus D Grassi W. Is the intima-media thickness of temporal and axillary arteries influenced by cardiovascular risk? Rheumatology. (2021) 60:5362–8. 10.1093/rheumatology/keab117 33547776

[19] Fernández-Fernández E Monjo-Henry I Bonilla G Plasencia C Miranda-Carús ME Balsa A. False positives in the ultrasound diagnosis of giant cell arteritis: some diseases can also show the halo sign. Rheumatology. (2020) 59:2443–7. 10.1136/annrheumdis-2019-eular.4008 31953951

[20] Molina Collada J Martínez-Barrio J Serrano-Benavente B Castrejón I Caballero Motta LR Trives Folguera L. Diagnostic value of ultrasound halo count and Halo Score in giant cell arteritis: a retrospective study from routine care. Ann Rheum Dis. (2020) 81:e175. 10.1136/annrheumdis-2020-218631 32759266

[21] Schmidt WA Natusch A Möller DE Vorpahl K Gromnica-Ihle E. Involvement of peripheral arteries in giant cell arteritis: a color Doppler sonography study. Clin Exp Rheumatol. (2002) 20:309–18. 12102466

[22] Schmidt WA Seifert A Gromnica-Ihle E Krause A Natusch A. Ultrasound of proximal upper extremity arteries to increase the diagnostic yield in large-vessel giant cell arteritis. Rheumatology. (2008) 47:96–101. 10.1093/rheumatology/kem322 18077499

[23] Agard C Hamidou MA Said L Ponge T Connault J Chevalet P. [Screening of abdominal aortic involvement using Doppler sonography in active giant cell (temporal) arteritis at the time of diagnosis. A prospective study of 30 patients]. La Revue de Medecine Interne. (2007) 28:363–70. 10.1016/j.revmed.2006.12.018 17275968

[24] Hop H Mulder DJ Sandovici M Glaudemans A van Roon AM Slart R. Diagnostic value of axillary artery ultrasound in patients with suspected giant cell arteritis. Rheumatology. (2020) 59:3676–84. 10.1093/rheumatology/keaa102 32240306 PMC7733725

[25] Nielsen BD Hansen IT Keller KK Therkildsen P Gormsen LC Hauge EM. Diagnostic accuracy of ultrasound for detecting large-vessel giant cell arteritis using FDG PET/CT as the reference. Rheumatology. (2020) 59:2062–73. 10.1093/rheumatology/kez568 31808526

[26] Gehlen M Schaefer N Schwarz-Eywill M Maier A. Ultrasound to detect involvement of vertebral artery in giant cell arteritis. Clin Exp Rheumatol. (2018) 36( Suppl 111):169–70. 29352850

[27] Czihal M Zanker S Rademacher A Tatò F Kuhlencordt PJ Schulze-Koops H. Sonographic and clinical pattern of extracranial and cranial giant cell arteritis. Scand J Rheumatol. (2012) 41:231–6. 10.3109/03009742.2011.641581 22400812

[28] Ješe R Rotar Ž Tomšič M Hočevar A. The role of colour doppler ultrasonography of facial and occipital arteries in patients with giant cell arteritis: a prospective study. Eur J Radiol. (2017) 95:9–12. 10.1016/j.ejrad.2017.07.007 28987704

[29] Diamantopoulos AP Haugeberg G Hetland H Soldal DM Bie R Myklebust G. Diagnostic value of color Doppler ultrasonography of temporal arteries and large vessels in giant cell arteritis: a consecutive case series. Arthritis Care Res. (2014) 66:113–9. 10.1002/acr.22178 24106211

[30] Zachrisson H Svensson C Dremetsika A Eriksson P. An extended high-frequency ultrasound protocol for detection of vessel wall inflammation. Clin Physiol Funct Imaging. (2018) 38:586–94. 10.1111/cpf.12450 28795494

[31] Karassa FB Matsagas MI Schmidt WA Ioannidis JP. Meta-analysis: test performance of ultrasonography for giant-cell arteritis. Ann Intern Med. (2005) 142:359–69. 10.7326/0003-4819-142-5-200503010-00011 15738455

[32] Arida A Kyprianou M Kanakis M Sfikakis PP. The diagnostic value of ultrasonography-derived edema of the temporal artery wall in giant cell arteritis: a second meta-analysis. BMC Musculoskelet Dis. (2010) 11:44. 10.1186/1471-2474-11-44 20210989 PMC2837862

[33] Ball EL Walsh SR Tang TY Gohil R Clarke JM. Role of ultrasonography in the diagnosis of temporal arteritis. Br J Surg. (2010) 97:1765–71. 10.1002/bjs.7252 20799290

[34] Seitz L Christ L Lötscher F Scholz G Sarbu AC Bütikofer L. Quantitative ultrasound to monitor the vascular response to tocilizumab in giant cell arteritis. Rheumatology. (2021) 60:5052–9. 10.1093/rheumatology/keab484 34117737 PMC8566271

[35] Soares C Costa A Santos R Abreu P Castro P Azevedo E. Clinical, laboratory and ultrasonographic interrelations in giant cell arteritis. J Stroke Cerebrovasc Dis. (2021) 30:105601. 10.1016/j.jstrokecerebrovasdis.2021.105601 33497936

[36] Sebastian A Tomelleri A Kayani A Prieto-Pena D Ranasinghe C Dasgupta B. Probability-based algorithm using ultrasound and additional tests for suspected GCA in a fast-track clinic. RMD Open. (2020) 6:e001297. 10.1136/rmdopen-2020-001297 32994361 PMC7547539

[37] Lauwerys BR Puttemans T Houssiau FA Devogelaer JP. Color Doppler sonography of the temporal arteries in giant cell arteritis and polymyalgia rheumatica. J Rheumatol. (1997) 24:1570–4. 9263153

[38] Schmid R Hermann M Yannar A Baumgartner RW. [Color duplex ultrasound of the temporal artery: replacement for biopsy in temporal arteritis]. Ophthalmologica. (2002) 216:16–21. 10.1159/000048291 11901283

[39] Karahaliou M Vaiopoulos G Papaspyrou S Kanakis MA Revenas K Sfikakis PP. Colour duplex sonography of temporal arteries before decision for biopsy: a prospective study in 55 patients with suspected giant cell arteritis. Arthritis Res Ther. (2006) 8:R116. 10.1186/ar2003 16859533 PMC1779378

[40] Santoro L D'Onofrio F Bernardi S Gremese E Ferraccioli G Santoliquido A. Temporal ultrasonography findings in temporal arteritis: early disappearance of halo sign after only 2 days of steroid treatment. Rheumatology. (2013) 52:622. 10.1093/rheumatology/kes387 23300333

[41] Pérez López J Solans Laqué R Bosch Gil JA Molina Cateriano C Huguet Redecilla P Vilardell Tarrés M. Colour-duplex ultrasonography of the temporal and ophthalmic arteries in the diagnosis and follow-up of giant cell arteritis. Clin Exp Rheumatol. (2009) 27(1 Suppl 52):S77–82. 19646351

[42] Aschwanden M Kesten F Stern M Thalhammer C Walker UA Tyndall A. Vascular involvement in patients with giant cell arteritis determined by duplex sonography of 2x11 arterial regions. Ann Rheum Dis. (2010) 69:1356–9. 10.1136/ard.2009.122135 20498213

[43] Diamantopoulos AP Myklebust G. Long-term inflammation in the temporal artery of a giant cell arteritis patient as detected by ultrasound. Ther Adv Musculoskelet Dis. (2014) 6:102–3. 10.1177/1759720X14521109 24891881 PMC4040938

[44] Ford JA DiIorio MA Huang W Sobiesczcyk P Docken WP Tedeschi SK. Follow-up vascular ultrasounds in patients with giant cell arteritis. Clin Exp Rheumatol. (2020) 38(Suppl 124):107–11. 32359038 PMC7812681

[45] De Miguel E Roxo A Castillo C Peiteado D Villalba A Martín-Mola E. The utility and sensitivity of colour Doppler ultrasound in monitoring changes in giant cell arteritis. Clin Exp Rheumatol. (2012) 30(1 Suppl 70):S34–8. 22410311

[46] Monti S Floris A Ponte CB Schmidt WA Diamantopoulos AP Pereira C. The proposed role of ultrasound in the management of giant cell arteritis in routine clinical practice. Rheumatology. (2018) 57:112–9. 10.1093/rheumatology/kex341 29045738

[47] Aschwanden M Schegk E Imfeld S Staub D Rottenburger C Berger CT. Vessel wall plasticity in large vessel giant cell arteritis: an ultrasound follow-up study. Rheumatology. (2019) 58:792–7. 10.1093/rheumatology/key383 30544199

[48] Ponte C Monti S Scirè CA Delvino P Khmelinskii N Milanesi A. Ultrasound halo sign as a potential monitoring tool for patients with giant cell arteritis: a prospective analysis. Ann Rheum Dis. (2021) 80:1475–82. 10.1136/annrheumdis-2021-220306 34215646

[49] Monti S Ponte C Pereira C Manzoni F Klersy C Rumi F. The impact of disease extent and severity detected by quantitative ultrasound analysis in the diagnosis and outcome of giant cell arteritis. Rheumatology (Oxford). (2020) 59:2299–307. 10.1093/rheumatology/kez554 31848610

[50] van der Geest KSM Borg F Kayani A Paap D Gondo P Schmidt W. Novel ultrasonographic Halo Score for giant cell arteritis: assessment of diagnostic accuracy and association with ocular ischaemia. Ann Rheum Dis. (2020) 79:393–9. 10.1136/annrheumdis-2019-216343 31900304 PMC7034352

[51] Schmidt WA Krause A Schicke B Kuchenbecker J Gromnica-Ihle E. Do temporal artery duplex ultrasound findings correlate with ophthalmic complications in giant cell arteritis? Rheumatology. (2009) 48:383–5. 10.1093/rheumatology/ken515 19179409

[52] Gribbons KB Ponte C Craven A Robson JC Suppiah R Luqmani R. Diagnostic assessment strategies and disease subsets in giant cell arteritis: data from an international observational cohort. Arthritis Rheumatol. (2020) 72:667–76. 10.1002/art.41165 31729185 PMC7113106

[53] Ponte C Serafim AS Monti S Fernandes E Lee E Singh S. Early variation of ultrasound halo sign with treatment and relation with clinical features in patients with giant cell arteritis. Rheumatology. (2020) 59:3717–26. 10.1093/rheumatology/keaa196 32393983

[54] Schmidt D Hetzel A Reinhard M Auw-Haedrich C. Comparison between color duplex ultrasonography and histology of the temporal artery in cranial arteritis (giant cell arteritis). Eur J Med Res. (2003) 8:1–7. 12578748

[55] Czihal M Piller A Schroettle A Kuhlencordt P Bernau C Schulze-Koops H. Impact of cranial and axillary/subclavian artery involvement by color duplex sonography on response to treatment in giant cell arteritis. J Vasc Surg. (2015) 61:1285–91. 10.1016/j.jvs.2014.12.045 25659455

[56] Bley TA Reinhard M Hauenstein C Markl M Warnatz K Hetzel A. Comparison of duplex sonography and high-resolution magnetic resonance imaging in the diagnosis of giant cell (temporal) arteritis. Arthritis Rheum. (2008) 58:2574–8. 10.1002/art.23699 18668559

[57] Ghinoi A Zuccoli G Nicolini A Pipitone N Macchioni L Bajocchi GL. 1T magnetic resonance imaging in the diagnosis of giant cell arteritis: comparison with ultrasonography and physical examination of temporal arteries. Clin Exp Rheumatol. (2008) 26(3 Suppl 49):S76–80. 18799059

[58] Hauenstein C Reinhard M Geiger J Markl M Hetzel A Treszl A. Effects of early corticosteroid treatment on magnetic resonance imaging and ultrasonography findings in giant cell arteritis. Rheumatology. (2012) 51:1999–2003. 10.1093/rheumatology/kes153 22772317

[59] Yip A Jernberg ET Bardi M Geiger J Lohne F Schmidt WA. Magnetic resonance imaging compared to ultrasonography in giant cell arteritis: a cross-sectional study. Arthritis Res Ther. (2020) 22:247. 10.1186/s13075-020-02335-4 33076985 PMC7574248

[60] Czihal M Tatò F Förster S Rademacher A Schulze-Koops H Hoffmann U. Fever of unknown origin as initial manifestation of large vessel giant cell arteritis: diagnosis by colour-coded sonography and 18-FDG-PET. Clin Exp Rheumatol. (2010) 28:549–52. 20659410

[61] Förster S Tato F Weiss M Czihal M Rominger A Bartenstein P. Patterns of extracranial involvement in newly diagnosed giant cell arteritis assessed by physical examination, colour coded duplex sonography and FDG-PET. VASA Zeitschrift fur Gefasskrankheiten. (2011) 40:219–27. 10.1024/0301-1526/a000096 21638250

[62] Imfeld S Aschwanden M Rottenburger C Schegk E Berger CT Staub D. [18F]FDG positron emission tomography and ultrasound in the diagnosis of giant cell arteritis: congruent or complementary imaging methods? Rheumatology. (2020) 59:772–8. 10.1093/rheumatology/kez362 31436837

[63] de Miguel E Andreu JL Naredo E Möller I. Ultrasound in rheumatology: where are we and where are we going? Reumatol Clin. (2014) 10:6–9. 10.1016/j.reuma.2013.04.005 23856277

[64] Mahr A Belhassen M Paccalin M Devauchelle-Pensec V Nolin M Gandon S. Characteristics and management of giant cell arteritis in France: a study based on national health insurance claims data. Rheumatology. (2020) 59:120–8. 10.1093/rheumatology/kez251 31382293

[65] Ing E Xu QA Chuo J Kherani F Landau K. Practice preferences: temporal artery biopsy versus doppler ultrasound in the work-up of giant cell arteritis. Neuro-ophthalmology. (2020) 44:174–81. 10.1080/01658107.2019.1656752 32395169 PMC7202440

[66] Diamantopoulos AP Haugeberg G Lindland A Myklebust G. The fast-track ultrasound clinic for early diagnosis of giant cell arteritis significantly reduces permanent visual impairment: towards a more effective strategy to improve clinical outcome in giant cell arteritis? Rheumatology. (2016) 55:66–70. 10.1093/rheumatology/kev289 26286743

[67] Chrysidis S Terslev L Christensen R Fredberg U Larsen K Lorenzen T. Vascular ultrasound for the diagnosis of giant cell arteritis: a reliability and agreement study based on a standardised training programme. RMD Open. (2020) 6:e001337. 10.1136/rmdopen-2020-001337 32978303 PMC7539855

[68] Sundholm JKM Paetau A Albäck A Pettersson T Sarkola T. Non-invasive vascular very-high resolution ultrasound to quantify artery intima layer thickness: validation of the four-line pattern. Ultrasound Med Biol. (2019) 45:2010–8. 10.1016/j.ultrasmedbio.2019.04.017 31101444

[69] Bergner R Splitthoff J Wadsack D. Use of contrast-enhanced ultrasound sonography in giant cell arteritis: a proof-of-concept study. Ultrasound Med Biol. (2022) 48:143–8. 10.1016/j.ultrasmedbio.2021.09.019 34702639

[70] Schinkel AF van den Oord SC van der Steen AF van Laar JA Sijbrands EJ. Utility of contrast-enhanced ultrasound for the assessment of the carotid artery wall in patients with Takayasu or giant cell arteritis. Eur Heart J Cardiovasc Imaging. (2014) 15:541–6. 10.1093/ehjci/jet243 24247923

[71] Germanò G Macchioni P Possemato N Boiardi L Nicolini A Casali M. Contrast-enhanced ultrasound of the carotid artery in patients with large vessel vasculitis: correlation with positron emission tomography findings. Arthritis Care Res. (2017) 69:143–9. 10.1002/acr.22906 27059104

[72] Roncato C Perez L Brochet-Guégan A Allix-Béguec C Raimbeau A Gautier G. Colour doppler ultrasound of temporal arteries for the diagnosis of giant cell arteritis: a multicentre deep learning study. Clin Exp Rheumatol. (2020) 38(Suppl 124):120–5. 32441644

[73] Dejaco C Ramiro S Duftner C Besson FL Bley TA Blockmans D. EULAR recommendations for the use of imaging in large vessel vasculitis in clinical practice. Ann Rheum Dis. (2018) 77:636–43. 10.1136/annrheumdis-2017-212649 29358285

[74] Mackie SL Dejaco C Appenzeller S Camellino D Duftner C Gonzalez-Chiappe S. British Society for Rheumatology guideline on diagnosis and treatment of giant cell arteritis: executive summary. Rheumatology. (2020) 59:487–94. 10.1093/rheumatology/kez664 31970410

[75] Pfadenhauer K Weinerth J Hrdina C. Vertebral arteries: a target for FDG-PET imaging in giant cell arteritis? Clinical, ultrasonographic and PET study in 46 patients. Nuklearmedizin. (2011) 50:28–32. 10.3413/nukmed-0335-10-07 21060976

